# Plasminogen activator inhibitor‐1 does not contribute to the pulmonary pathology induced by acute exposure to ozone

**DOI:** 10.14814/phy2.12983

**Published:** 2016-09-26

**Authors:** Hamza S. Elkhidir, Jeremy B. Richards, Kevin R. Cromar, Cynthia S. Bell, Roger E. Price, Constance L. Atkins, Chantal Y. Spencer, Farhan Malik, Amy L. Alexander, Katherine J. Cockerill, Ikram U. Haque, Richard A. Johnston

**Affiliations:** ^1^ Division of Critical Care MedicineDepartment of PediatricsMcGovern Medical School at The University of Texas Health Science Center at HoustonHoustonTexas; ^2^ Division of PulmonaryCritical Care and Sleep MedicineDepartment of MedicineCollege of MedicineMedical University of South CarolinaCharlestonSouth Carolina; ^3^ Department of Environmental MedicineNew York University School of MedicineTuxedoNew York; ^4^ Division of NephrologyDepartment of PediatricsMcGovern Medical School at The University of Texas Health Science Center at HoustonHoustonTexas; ^5^ Comparative Pathology LaboratoryCenter for Comparative MedicineBaylor College of MedicineHoustonTexas; ^6^ Division of Pulmonary MedicineDepartment of PediatricsMcGovern Medical School at The University of Texas Health Science Center at HoustonHoustonTexas; ^7^ Section of Pediatric PulmonologyDepartment of PediatricsBaylor College of MedicineHoustonTexas; ^8^ Pediatric Research CenterDepartment of PediatricsMcGovern Medical School at The University of Texas Health Science Center at HoustonHoustonTexas; ^9^ Department of Integrative Biology and PharmacologyMcGovern Medical School at The University of Texas Health Science Center at HoustonHoustonTexas

**Keywords:** Airway hyperresponsiveness, epithelial cell, lung injury, macrophage inflammatory protein‐2, neutrophil

## Abstract

Expression of plasminogen activator inhibitor (PAI)‐1, the major physiological inhibitor of fibrinolysis, is increased in the lung following inhalation of ozone (O_3_), a gaseous air pollutant. PAI‐1 regulates expression of interleukin (IL)‐6, keratinocyte chemoattractant (KC), and macrophage inflammatory protein (MIP)‐2, which are cytokines that promote lung injury, pulmonary inflammation, and/or airway hyperresponsiveness following acute exposure to O_3_. Given these observations, we hypothesized that PAI‐1 contributes to the severity of the aforementioned sequelae by regulating expression of IL‐6, KC, and MIP‐2 following acute exposure to O_3_. To test our hypothesis, wild‐type mice and mice genetically deficient in PAI‐1 (PAI‐1‐deficient mice) were acutely exposed to either filtered room air or O_3_ (2 ppm) for 3 h. Four and/or twenty‐four hours following cessation of exposure, indices of lung injury [bronchoalveolar lavage fluid (BALF) protein and epithelial cells], pulmonary inflammation (BALF IL‐6, KC, MIP‐2, macrophages, and neutrophils), and airway responsiveness to aerosolized acetyl‐*β*‐methylcholine chloride (respiratory system resistance) were measured in wild‐type and PAI‐1‐deficient mice. O_3_ significantly increased indices of lung injury, pulmonary inflammation, and airway responsiveness in wild‐type and PAI‐1‐deficient mice. With the exception of MIP‐2, which was significantly lower in PAI‐1‐deficient as compared to wild‐type mice 24 h following cessation of exposure to O_3_, no other genotype‐related differences occurred subsequent to O_3_ exposure. Thus, following acute exposure to O_3_, PAI‐1 neither regulates pulmonary expression of IL‐6 and KC nor functionally contributes to any of the pulmonary pathological sequelae that arise from the noxious effects of inhaled O_3_.

## Introduction

Tropospheric ozone (O_3_) is a gaseous air pollutant and a powerful oxidant generated from photochemical reactions involving nitrogen oxides and volatile organic compounds (Finlayson‐Pitts and Pitts 1997). Once inhaled into the lungs, O_3_ rapidly reacts with proteins and unsaturated fatty acids in the airway surface liquid to initiate a cascade of events that results in lung injury, pulmonary inflammation, and airway hyperresponsiveness (AHR) (United States Environmental Protection Agency, 2013). O_3_‐induced lung injury is characterized, in part, by desquamation of airway epithelial cells and hyperpermeability of the alveolar‐capillary membrane (Scheel et al. 1959; Bhalla et al. 1986). Pulmonary inflammation induced by exposure to O_3_ is typified by increased expression of inflammatory cytokines [interleukin (IL)‐1*α*, IL‐1*β*, IL‐6, IL‐17A, keratinocyte chemoattractant (KC), macrophage inflammatory protein (MIP)‐2, osteopontin, and tumor necrosis factor (TNF)] in lung tissue and/or bronchoalveolar lavage fluid (BALF) and by migration of macrophages and neutrophils to air spaces (Park et al. 2004; Johnston et al. 2005a,b; Barreno et al. 2013; Kasahara et al. 2015; Razvi et al. 2015). O_3_ also causes AHR to nonspecific bronchoconstrictors, which is a phenomenon dependent on many of the inflammatory cytokines whose expression is induced by O_3_ (Golden et al. 1978; Seltzer et al. 1986; Foster et al. 2000; Cho et al. 2001; Shore et al. 2001; Park et al. 2004; Johnston et al. 2005a; Lu et al. 2006; Barreno et al. 2013).

Elevations in ambient O_3_ increase hospitalizations for individuals with respiratory disease (Medina‐Ramón et al. 2006; Silverman and Ito 2010). O_3_ also disproportionally increases mortality among the elderly and among people with respiratory disease (Bell et al. 2014; Hao et al. 2015). Because these vulnerable subpopulations continue to increase in the United States (U.S.) and because 51% of the U.S. population lives in areas with unhealthy levels of O_3_ in ambient air (Administration on Aging; Wroe et al. 2012; Zhang et al. 2013; American Lung Association, 2016), a significant number of individuals within these subpopulations are susceptible to the harmful effects of O_3_. Thus, to mitigate or prevent O_3_‐induced lung dysfunction and even possible death in vulnerable individuals, it is imperative to understand the molecular mechanisms underlying O_3_‐induced pulmonary pathology.

Ozone increases pulmonary expression of plasminogen activator inhibitor (PAI)‐1, a single‐chain 50 kDa glycoprotein (van Mourik et al. 1984; Katre et al. 2011; Kodavanti et al. 2011). However, the functional consequences of this phenomenon are not presently known. PAI‐1 is a member of the serpin superfamily of protease inhibitors and is expressed by a number of cells, including adipocytes, endothelial cells, epithelial cells, fibroblasts, mast cells, and platelets (van Hinsbergh et al. 1988; Gerwin et al. 1990; Konkle et al. 1993; Samad et al. 1994; Samad and Loskutoff 1996; Cho et al. 2000; Heit et al. 2013). PAI‐1 is the major physiological inhibitor of tissue‐type plasminogen activator (t‐PA) and urokinase‐type plasminogen activator (u‐PA) (Van De Craen et al. 2012). Because t‐PA and u‐PA activate the fibrinolytic system by catalyzing the conversion of plasminogen to plasmin, PAI‐1 is commonly regarded as the primary inhibitor of fibrinolysis (Flemmig and Melzig 2012; Van De Craen et al. 2012). Structurally, PAI‐1 can exist in several confirmations, including active, latent, and in complex with either t‐PA or u‐PA (Munch et al. 1993; Aertgeerts et al. 1995; Grebenschikov et al. 1999; Van De Craen et al. 2012). The active form of PAI‐1 is capable of forming covalent complexes with t‐PA or u‐PA, and thus, inhibit the ability of t‐PA and u‐PA to initiate the fibrinolysis cascade (Van De Craen et al. 2012). The latent form of PAI‐1 appears to have no functional capability (Van De Craen et al. 2012).

In addition to inhibiting fibrinolysis, PAI‐1 exerts other biological effects that suggest PAI‐1 may contribute to the severity of the pathological features observed in the lung following acute exposure to O_3_. First, in several diverse animal models of lung disease, PAI‐1 is necessary for migration of macrophages and neutrophils to air spaces, development of pulmonary vascular hyperpermeability, and manifestation of AHR (Arndt et al. 2005, 2006; Kuramoto et al. 2009; Goolaerts et al. 2011; Wolthuis et al. 2011; Lee et al. 2012; Bhandary et al. 2015; Tezuka et al. 2015; Liu et al. 2016), which are all pathological features induced by inhalation of O_3_ (Bhalla et al. 1986; Foster et al. 2000; Johnston et al. 2005a; Razvi et al. 2015). Second, within the lung, PAI‐1 is necessary for the maximal expression of specific cytokines (IL‐6, KC, MIP‐2, and TNF) and chemokine (C‐X‐C motif) receptor 2 (CXCR2) (Renckens et al. 2007; Wolthuis et al. 2011; Tiwari et al. 2016), the receptor for KC and MIP‐2 (Konrad and Reutershan 2012). CXCR2 and these aforementioned cytokines elicit desquamation of airway epithelial cells, migration of neutrophils to air spaces, and/or AHR following O_3_ exposure (Cho et al. 2001; Shore et al. 2001; Johnston et al. 2005a,b; Lang et al. 2008). Third, expression of PAI‐1 in the lung is increased by c‐Jun N‐terminal kinase (Arndt et al. 2005), which contributes to O_3_‐induced pulmonary inflammation and O_3_‐induced AHR (Williams et al. 2007). Given these observations, we hypothesized that PAI‐1 contributes to the severity of pulmonary pathological features induced by acute exposure to O_3_.

To test our hypothesis, wild‐type mice and mice genetically deficient in PAI‐1 (PAI‐1‐deficient mice) were acutely exposed to either filtered room air (air) or O_3_ [2 parts/million (ppm)] for 3 h. Four and twenty‐four hours following cessation of exposure, we assessed indices of lung injury and pulmonary inflammation in wild‐type and PAI‐1‐deficient mice by biochemically or histologically analyzing bronchoalveolar lavage fluid (BALF) supernatants and cell differentials and formalin‐fixed and paraffin‐embedded lung sections. The forced oscillation technique was used to assess airway responsiveness to aerosolized acetyl‐*β*‐methylcholine chloride (methacholine) in wild‐type and PAI‐1‐deficient mice 24 h following cessation of exposure. Consistent with other investigators (Katre et al. 2011; Kodavanti et al. 2011), our data demonstrate that O_3_ increases PAI‐1 in the lungs. However, our data also reveal that PAI‐1 does not functionally contribute to any aspect of the pulmonary pathology induced by acute exposure to O_3_.

## Materials and Methods

### Animals

Mice homozygous for a null mutation in the gene encoding PAI‐1 (PAI‐1‐deficient mice) were generated *via* homologous recombination as previously described by Carmeliet et al. (Carmeliet et al. 1993). Female PAI‐1‐deficient mice were purchased from The Jackson Laboratory (Bar Harbor, ME) or generated in a multi‐species modified barrier animal care facility at McGovern Medical School at The University of Texas Health Science Center at Houston (Houston, TX) from either mating pairs or trios of PAI‐1‐deficient mice that were also purchased from The Jackson Laboratory. Because PAI‐1‐deficient mice were backcrossed into a C57BL/6J background for 10 generations (The Jackson Laboratory), age‐matched female C57BL/6J mice were purchased from The Jackson Laboratory and used as wild‐type controls. The care and use of all animals in this study adhered to the guidelines of the National Institutes of Health (Bethesda, MD) while each of the experimental protocols used in this study were approved by the Animal Welfare Committee of The University of Texas Health Science Center at Houston (Houston, TX).

### Protocol

A number of experiments were performed in this study that required three separate cohorts of wild‐type and PAI‐1‐deficient mice. In the first cohort, mice were euthanized 4 or 24 h following cessation of a 3 h exposure to either air or O_3_ (2 ppm). Blood and BALF were subsequently obtained from these animals. Mice in the second cohort were euthanized 24 h following cessation of a 3 h exposure to either air or O_3_ (2 ppm). Afterwards, blood was collected from each animal and the lungs fixed in situ and removed from the thoracic cavity of the animal *en bloc*. In the third cohort, the animals were anesthetized 24 h following cessation of a 3 h exposure to either air or O_3_ (2 ppm), and airway responsiveness to aerosolized methacholine was measured.

### Air and O_3_ exposure

Conscious mice were individually placed into one of eight cells of a stainless steel wire mesh cage (Marlin Steel Wire Products LLC, Baltimore, MD) that was subsequently placed inside a powder‐coated aluminum exposure chamber with a Plexiglas^®^ door (Teague Enterprises; Woodland, CA). Once the Plexiglas^®^ door of the chamber was securely closed, the animals were exposed to either air or O_3_ (2 ppm) for 3 h. After the 3 h exposure was complete, the animals were placed into the same micro‐isolator cage (TECNIPLAST S.p.A.; Buguggiate, Varese, Italy) that they occupied prior to air or O_3_ exposure. Once the mice were returned to the micro‐isolator cage, they had access to food and water ad libitum until the experimental procedures described below were performed 4 h and/or 24 h following cessation of exposure. For greater details with regard to air and O_3_ exposures, please refer to a prior publication from our laboratory (Razvi et al. 2015).

### Blood withdrawal

Mice in the first and second cohorts were euthanized with an intraperitoneal (i.p.) injection of pentobarbital sodium (200 mg/kg; Vortech Pharmaceuticals, Ltd.; Dearborn, MI). Once each animal was deeply anesthetized, a median thoracotomy was performed to expose the heart and lungs in situ. The right ventricle of the heart was punctured with a 25‐gauge needle attached to a 1 mL syringe, and blood was slowly withdrawn from the heart. Blood was removed from the heart, and by extension, the pulmonary circulation, in order to prevent blood from contaminating the BALF and interfering with the immunohistochemical staining of lung tissue.

### BAL

A BAL was performed on each animal within the first cohort after blood was withdrawn from the heart. BALF was subsequently collected, processed, and stored until needed. In addition, the total number of BALF cells was enumerated, and a differential count of BALF cells was performed. Approximately 80% of the instilled lavage buffer was retrieved from each animal, and there was no effect of genotype or exposure on the volume of lavage buffer that was retrieved. A detailed description of the collection, processing, and storage of BALF and of the enumeration of total BALF cells and differentials have been previously described by our laboratory (Razvi et al. 2015). Finally, within each genotype, there were no significant differences between the BALF indices measured at four as compared to 24 h following cessation of exposure to air. Thus, data for all air‐exposed and genotype‐matched mice were pooled (data not shown).

### Immunoassays and protein quantification

The concentration of BALF IL‐6, KC, MIP‐2, and total and active PAI‐1 were measured using enzyme‐linked immunosorbent assays (R&D Systems, Inc., Minneapolis, MN for IL‐6, KC, and MIP‐2 and Molecular Innovations, Inc., Novi, MI for total and active PAI‐1) according to the manufacturer's instructions. BALF protein was quantified using the Bradford protein assay as previously described by Bradford (1976); Razvi et al. (2015).

### Lung histology and immunohistochemistry

After blood was withdrawn from the heart of each animal within the second cohort, the heart and entire circulation were flushed with 10 mL of precooled phosphate‐buffered saline (PBS). Subsequently, the lungs were fixed in situ with 10% phosphate‐buffered formalin (Fisher Scientific, Fair Lawn, NJ) at a pressure of 25 cm H_2_O. Lungs were then dehydrated and paraffin‐embedded. Sections were cut from paraffin‐embedded lungs and mounted onto glass microscope slides. Afterwards, the sections were either stained with hematoxylin and eosin or an anti‐mouse PAI‐1 polyclonal antibody (1 μg/mL; Abcam plc., Cambridge, MA) and examined *via* bright‐field microscopy by a veterinary pathologist according to previously published procedures (Dahm et al. 2014; Razvi et al. 2015). Peribronchiolar and perivascular inflammation were also scored in hematoxylin‐ and eosin‐stained lung sections as previously described (Dahm et al. 2014; Razvi et al. 2015).

### Measurement of respiratory system responsiveness to methacholine

Mice in the third cohort were anesthetized with pentobarbital sodium (50 mg/kg, i.p.; Oak Pharmaceuticals, Inc., Lake Forest, IL) and xylazine hydrochloride (7 mg/kg, i.p.; Vedco Inc., St. Joseph, MO), instrumented for mechanical ventilation, and ventilated at a frequency of 2.5 Hz, a tidal volume of 0.3 mL, and a positive end‐expiratory pressure of 3 cm H_2_O using a specialized ventilator that is capable of measuring indices of respiratory system mechanics using the forced oscillation technique (*flexiVent*; SCIREQ Scientific Respiratory Equipment Inc.; Montréal, Québec, Canada) (Schuessler and Bates 1995). The *flexiVent* was used in this study to measure responses to aerosolized PBS followed by increasing doses of aerosolized methacholine (0.1–100 mg/mL) to determine respiratory system resistance (*R*_RS_). For each animal in the third cohort, we also calculated the dose of methacholine required to double *R*_RS_ (ED_200_ *R*_RS_). The ED_200_ *R*_RS_ was calculated by logarithmic‐linear interpolation between the doses abutting the point at which *R*_RS_ was exactly 200% of the baseline *R*_RS_ value, which was defined as the *R*_RS_ value obtained following PBS administration. All measurements were made in animals with a closed chest. For a more detailed description of the methods we used to measure respiratory system resistance to methacholine in this study, please refer to Razvi et al. (2015).

### Statistical analyses of data

The effect of genotype (wild‐type or PAI‐1‐deficient) and exposure (air or O_3_) on BALF indices, peribronchiolar and perivascular inflammation, baseline *R*_RS_, and the logarithm of ED_200_ *R*_RS_ were assessed by a two‐way analysis of variance (ANOVA) for normally distributed data or by a Kruskal–Wallis one‐way ANOVA for non‐normally distributed data. Depending on whether the data were normally or non‐normally distributed, the Fisher's least significant difference test or the Conover‐Iman test with a Bonferroni adjustment, respectively, were used for *post hoc* analyses to determine the significance of differences between groups. Statistical analysis of the repeated measures comprising the methacholine dose–response curves was completed using the area under the curve (AUC) analysis with respect to increased response compared with the response following PBS administration. AUC analysis was performed using R (Version 2.15.3) (R Core Team 2013). Stata 12 was used for all other statistical analyses (StataCorp LP, College Station, TX). Unless otherwise noted, the results are expressed as the mean ± the standard error of the mean. A *P* value less than 0.05 was considered significant.

## Results

### Effect of PAI‐1 deficiency and O_3_ on BALF total and active PAI‐1

**Figure 1:**
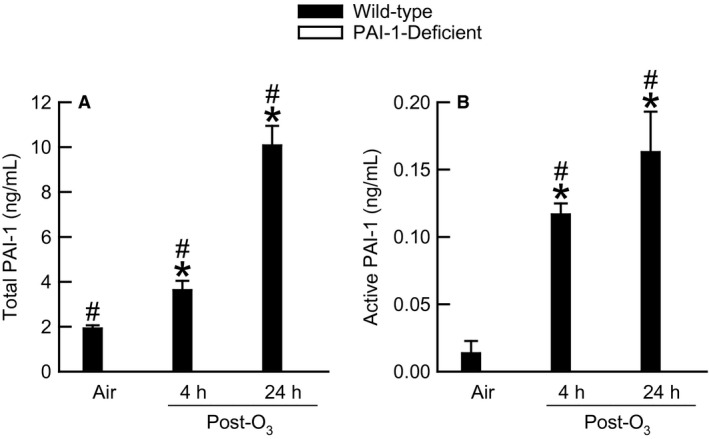
The concentration of (A) total and (B) active plasminogen activator inhibitor (PAI)‐1 in bronchoalveolar lavage fluid (BALF) obtained from wild‐type C57BL/6 and mice genetically deficient in PAI‐1 (PAI‐1‐deficient mice) 4 and 24 h following cessation of a 3 h exposure to either filtered room air (air) or ozone [O_3_; 2 ppm]. Neither total nor active PAI‐1 was detectable in BALF of PAI‐1‐deficient mice exposed to either air or O_3_ when using the immunoassays described in Materials and Methods. Each value is the mean ± the standard error of the mean. *n *=* *6–10 mice in each group. *P <* *0.05 compared to genotype‐matched mice exposed to air. ^#^ *P *<* *0.05 compared to PAI‐1‐deficient mice with an identical exposure.

Using commercially available immunoassays (Molecular Innovations, Inc.), we measured the concentration of total and active PAI‐1 in BALF obtained from wild‐type and PAI‐1‐deficient mice 4 and 24 h following cessation of exposure to either air or O_3_ (Fig. 1). The total PAI‐1 immunoassay detects active and latent PAI‐1 and PAI‐1 in complex with t‐PA (David S. Ginsberg, M.S., pers. comm., of Molecular Innovations, Inc.). The total PAI‐1 immunoassay cannot determine the precise proportion of each PAI‐1 confirmation present in BALF. However, regardless of its confirmation, PAI‐1 was detectable in BALF obtained from wild‐type mice exposed to air (Fig. 1A). Four and twenty‐four hours following cessation of exposure to O_3_, there were significant twofold and fivefold increases, respectively, in BALF total PAI‐1 (Fig. 1A). We did attempt to measure total PAI‐1 in BALF of air‐ or O_3_‐exposed PAI‐1‐deficient mice using the aforementioned immunoassay, yet PAI‐1 was not detectable in any BALF obtained from PAI‐1‐deficient mice. We also measured the amount of active PAI‐1 in BALF obtained from wild‐type and PAI‐1‐deficient mice exposed to air or O_3_ (Fig. 1B), and the results were qualitatively similar to those obtained for total PAI‐1. Active PAI‐1 comprised less than two percent of the total PAI‐1 present in BALF.

### Immunostaining of PAI‐1 in lungs of air‐ or O_3_‐exposed wild‐type mice

**Figure 2:**
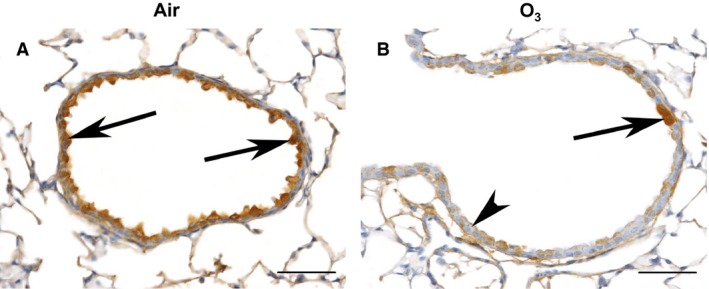
Representative light photomicrographs demonstrating the presence of plasminogen activator inhibitor (PAI)‐1 immunostaining within bronchiolar epithelial cells of wild‐type C57BL/6 mice 24 h following cessation of a 3 h exposure to either (A) filtered room air (air) or (B) ozone (O_3_; 2 ppm). The arrows in A and B are directed at bronchiolar epithelial cells that are strongly positive for PAI‐1 while the arrowhead in B is directed at a bronchiolar epithelial cell that is weakly positive for PAI‐1. Tissue sections in A and B were incubated with an anti‐mouse PAI‐1 polyclonal antibody. The lungs were fixed in situ with 10% phosphate‐buffered formalin 24 h following cessation of exposure to either air or O_3_. The images in A and B have been magnified with a 40× objective lens while each of the scale bars in A and B represent 50 μm. *n *=* *6 mice in each group.

Immunohistochemistry was used to identify the cells in the lungs of wild‐type mice that express PAI‐1 24 h following exposure to either air or O_3_ (Fig. 2). We chose to examine PAI‐1 immunostaining in the lungs of wild‐type mice 24 h following cessation of exposure because we observed the greatest increase in BALF PAI‐1 by O_3_ at this time (Fig. 1). As shown in Figure 2A, the apical cytoplasm of bronchiolar epithelial cells in air‐exposed wild‐type mice was strongly positive for PAI‐1. In contrast to the greater amount of PAI‐1 detected in BALF of wild‐type mice exposed to O_3_ (Fig. 1), the intensity of PAI‐1 immunostaining in the bronchiolar epithelium was less consistent and much weaker in wild‐type mice exposed to O_3_ as compared to air (Fig. 2B).

### Effect of PAI‐1 deficiency on pulmonary inflammation induced by O_3_

**Figure 3:**
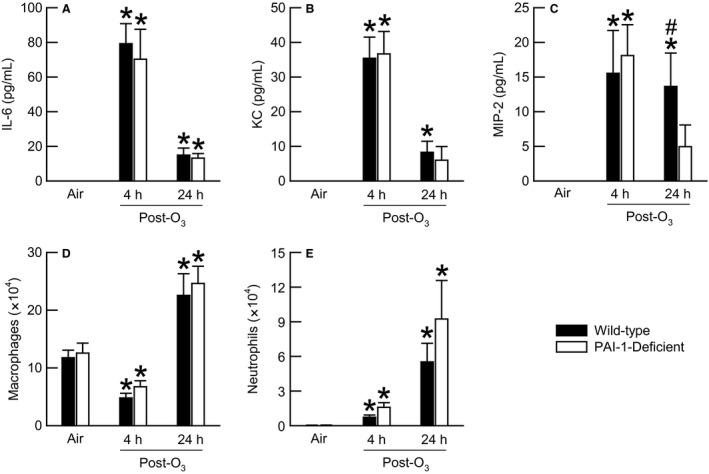
The concentration of (A) interleukin (IL)‐6, (B) keratinocyte chemoattractant (KC), and (C) macrophage inflammatory protein (MIP)‐2 as well as the number of (D) macrophages and (E) neutrophils in bronchoalveolar lavage fluid (BALF) obtained from wild‐type C57BL/6 mice and mice genetically deficient in PAI‐1 (PAI‐1‐deficient mice) 4 and 24 h following cessation of a 3 h exposure to either filtered room air (air) or ozone (O_3_; 2 ppm). IL‐6, KC, and MIP‐2 were not detectable in BALF of wild‐type and PAI‐1‐deficient mice exposed to air when using the immunoassays described in Materials and Methods. Each value is the mean ± the standard error of the mean. *n *=* *6–10 mice in each group. *P <* *0.05 compared to genotype‐matched mice exposed to air. ^#^ *P *<* *0.05 compared to PAI‐1‐deficient mice with an identical exposure.

Plasminogen activator inhibitor‐1 can regulate pulmonary expression of IL‐6, KC, and MIP‐2, which are necessary for desquamation of airway epithelial cells, migration of neutrophils to air spaces, and/or development of AHR following acute exposure to O_3_ (Johnston et al. 2005a,b; Renckens et al. 2007; Lang et al. 2008; Wolthuis et al. 2011; Tiwari et al. 2016). In addition, PAI‐1 regulates migration of macrophages to air spaces, which is a characteristic feature of O_3_‐induced pulmonary pathology (Bhandary et al. 2015; Razvi et al. 2015). Therefore, we measured BALF IL‐6, KC, and MIP‐2 and enumerated the number of BALF macrophages and neutrophils 4 and 24 h following cessation of exposure to air or O_3_ in order to assess the effect of PAI‐1 deficiency on development of O_3_‐induced pulmonary inflammation (Fig. 3). In air‐exposed wild‐type and PAI‐1‐deficient mice, IL‐6, KC, and MIP‐2 were not detectable in BALF (Fig. 3A–C). Four hours following cessation of exposure to O_3_, IL‐6, KC, and MIP‐2 were significantly increased in BALF of wild‐type and PAI‐1‐deficient mice (Fig. 3A–C). However, we observed no genotype‐related differences in any of these cytokines at this time interval. Twenty‐four hours following cessation of exposure to O_3_, BALF IL‐6, KC, and MIP‐2 in wild‐type and PAI‐1‐deficient mice still remained higher than those levels observed in genotype‐matched air‐exposed controls (Fig. 3A–C). With the exception of MIP‐2, which was significantly lower in PAI‐1‐deficient as compared to wild‐type mice, there were no other genotype‐related differences in cytokine levels 24 h following cessation of exposure to O_3_ (Fig. 3A–C).

Compared to genotype‐matched air‐exposed mice, there was a significant decrease in the number of BALF macrophages 4 h following O_3_ exposure but a significant increase in the number of BALF macrophages at 24 h following O_3_ exposure (Fig. 3D). However, we observed no genotype‐related differences in the number of BALF macrophages following air or O_3_ exposure. Irrespective of whether wild‐type or PAI‐1‐deficient mice were examined 4 or 24 h following cessation of exposure to O_3_, O_3_ caused a significant increase in the number of BALF neutrophils in both genotypes (Fig. 3E). However, there were no genotype‐related differences in the number of BALF neutrophils following air or O_3_ exposure.

**Figure 4:**
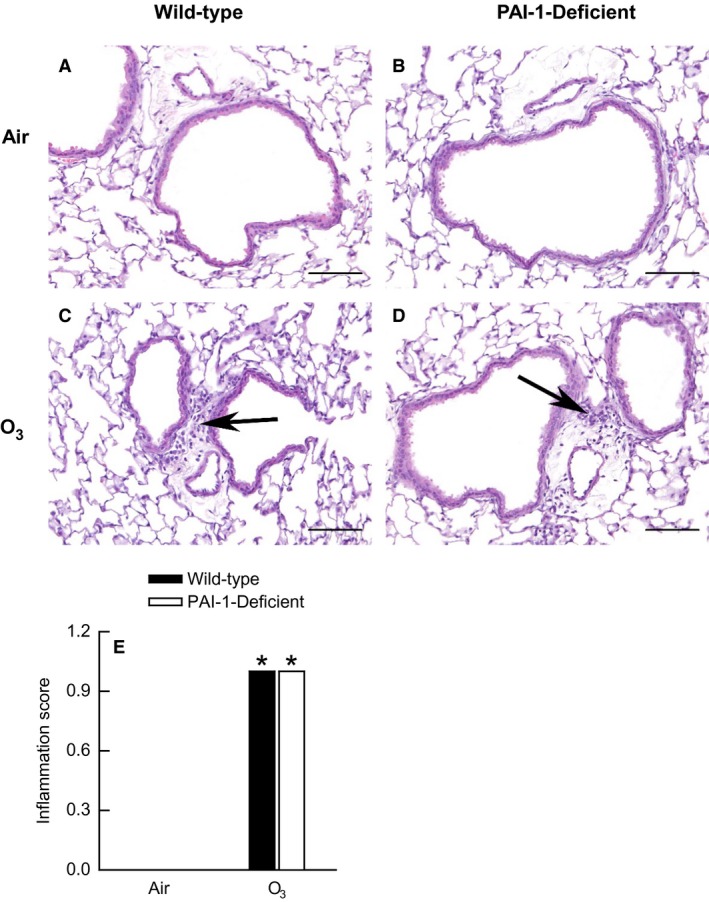
(A–D) Representative light photomicrographs of hematoxylin‐ and eosin‐stained lung sections and (E) lung perivascular inflammation scores from wild‐type C57BL/6 mice and mice genetically deficient in plasminogen activator inhibitor (PAI)‐1 (PAI‐1‐deficient mice) 24 h following cessation of a 3 h exposure to either filtered room air (air) or ozone (O_3_; 2 ppm). (A) and (B) are lung sections from air‐exposed wild‐type and PAI‐1‐deficient mice, respectively. (C) and (D) are lung sections from O_3_‐exposed wild‐type and PAI‐1‐deficient mice, respectively. The arrows in (C) and (D) are directed at infiltrates of mononuclear cells and neutrophils in the vicinity of pulmonary blood vessels. The lungs were fixed in situ with 10% phosphate‐buffered formalin 24 h following cessation of exposure to air or O_3_. In (A–D), the images have been magnified with a 40× objective lens while each of the scale bars in (A–D) represent 50 μm. In (E), each value is expressed as the mean ± the standard error of the mean. *n *=* *6 mice for each group. *P <* *0.05 compared to genotype‐matched mice exposed to air.

To further assess pulmonary inflammation in wild‐type and PAI‐1‐deficient mice, we scored peribronchiolar and perivascular inflammation in hematoxylin‐ and eosin‐stained lung sections (Fig. 4 and data not shown). Since the number of BALF macrophages and neutrophils were highest at 24 h following cessation of exposure to O_3_ (Fig. 3D and E), we choose this time interval to assess peribronchiolar and perivascular inflammation. No inflammatory lesions were present in the lungs of air‐exposed wild‐type and PAI‐1‐deficient mice (Fig. 4A, B, and E). In mice of both genotypes, O_3_ caused mild, but significant, perivascular inflammation characterized by multifocal infiltrations of mononuclear cells and neutrophils (Fig. 4C–E). However, there were no genotype‐related differences in perivascular inflammation scores between wild‐type and PAI‐1‐deficient mice (Fig. 4E). No peribronchiolar inflammation was present in the lungs of O_3_‐exposed wild‐type and PAI‐1‐deficient mice (data not shown).

### Effect of PAI‐1 deficiency on lung injury induced by O_3_

**Figure 5:**
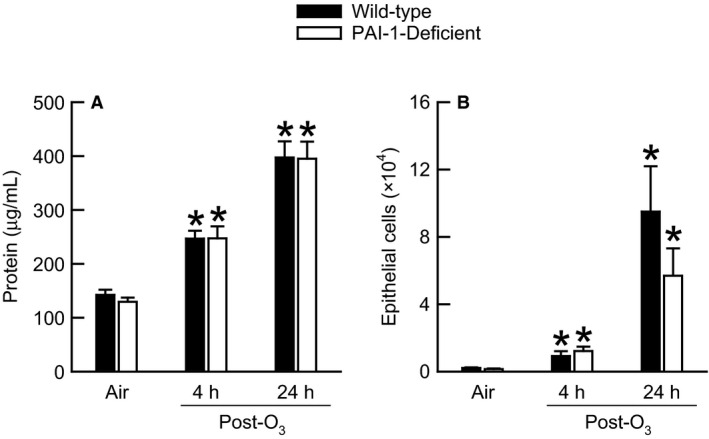
(A) The concentration of protein and (B) the number of epithelial cells in bronchoalveolar lavage fluid (BALF) obtained from wild‐type C57BL/6 and mice genetically deficient in PAI‐1 (PAI‐1‐deficient mice) 4 and 24 h following cessation of a 3 h exposure to either filtered room air (air) or ozone (O_3_; 2 ppm). Each value is the mean ± the standard error of the mean. *n *=* *6–10 mice in each group. *P <* *0.05 compared to genotype‐matched mice exposed to air.

Ozone causes lung injury characterized by pulmonary vascular hyperpermeability and desquamation of airway epithelial cells (Scheel et al. 1959; Bhalla et al. 1986). In response to injury, PAI‐1 regulates pulmonary vascular permeability and repair of alveolar epithelial cells (Lazar et al. 2004; Goolaerts et al. 2011). Thus, we measured BALF protein, which is an index of pulmonary vascular hyperpermeability (Alpert et al. 1971), and enumerated the number of BALF epithelial cells (Fig. 5). In both genotypes, O_3_ caused a significant increase in BALF protein 4 and 24 h following cessation of exposure when compared to genotype‐matched air‐exposed controls (Fig. 5A). However, no genotype‐related differences in BALF protein were observed at any time interval. Similar observations were made for BALF epithelial cells (Fig. 5B).

### Effect of PAI‐1 deficiency on O_3_‐induced AHR

**Figure 6:**
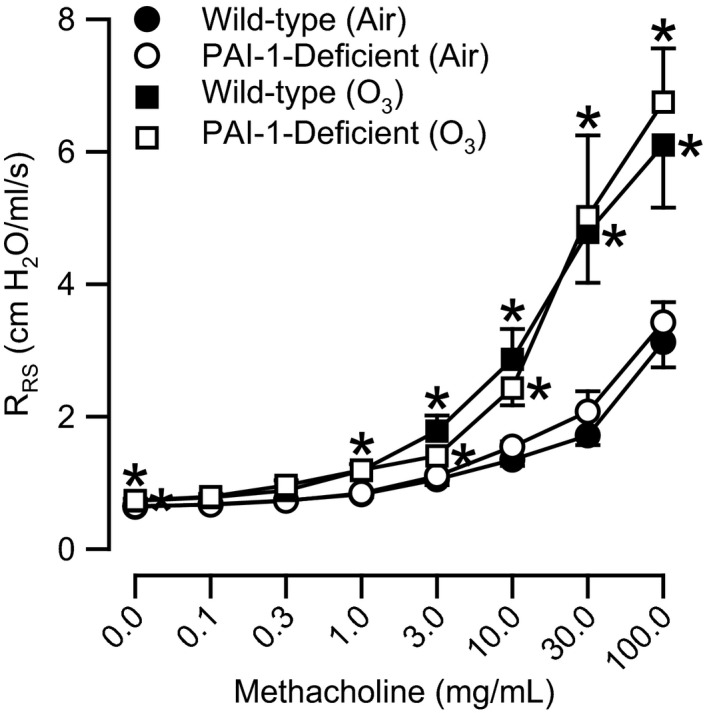
Responses to aerosolized acetyl‐*β*‐methylcholine chloride (methacholine) for respiratory system resistance (*R*_RS_) in wild‐type C57BL/6 mice and mice genetically deficient in plasminogen activator inhibitor (PAI)‐1 (PAI‐1‐deficient mice) 24 h following cessation of a 3 h exposure to either filtered room air (air) or ozone (O_3_; 2 ppm). Each value is the mean ± the standard error of the mean. *n *=* *6–9 mice in each group. *P <* *0.05 compared to genotype‐matched mice exposed to air.

In Table 1, we report *R*_RS_ values at baseline and values for ED_200_ *R*_RS_ that were obtained from wild‐type and PAI‐1‐deficient mice 24 h following cessation of exposure to either air or O_3_. Baseline *R*_RS_ values were not different between wild‐type and PAI‐1‐deficient mice following exposure to air. O_3_ significantly increased baseline *R*_RS_ in mice of both genotypes (Table 1 and Fig. 6). However, *R*_RS_ values at baseline were not different between wild‐type and PAI‐1‐deficient mice following O_3_ exposure (Table 1).

**Table 1:** Respiratory system resistance at baseline and effective dose of methacholine necessary to cause a 200% increase in baseline *R*_RS_ for wild‐type and PAI‐1‐deficient mice exposed to filtered room air or ozone

Genotype (Exposure)	*R*_RS_ (cm H_2_O/mL/sec)	ED_200_ *R*_RS_ (mg/mL) (95% Confidence interval)
Wild‐type (Air)	0.64 ± 0.02	6.8 (4.0–11.5)
PAI‐1‐Deficient (Air)	0.65 ± 0.01	6.1 (4.2–8.9)
Wild‐type (O_3_)	0.73 ± 0.03*	2.2* (1.0–4.8)
PAI‐1‐Deficient (O_3_)	0.73 ± 0.03*	3.1 (1.4–7.0)

Results are expressed as the mean ± the standard error of the mean (*R*_RS_, respiratory system resistance) or mean and 95% confidence interval (ED_200_ *R*_RS_, effective dose of methacholine necessary to cause a 200% increase in baseline *R*_RS_) from 6 to 9 mice in each group. *R*_RS_ measurements at baseline were made following the administration of phosphate‐buffered saline. *R*_RS_ at baseline and ED_200_ *R*_RS_ were measured or calculated, respectively, 24 h following cessation of exposure to filtered room air (air) or ozone (O_3_; 2 ppm) for 3 h. *P <* *0.05 compared to genotype‐matched mice exposed to air.

In air‐exposed wild‐type and PAI‐1‐deficient mice, methacholine caused significant increases in *R*_RS_ when compared to *R*_RS_ measurements obtained following PBS administration. However, no differences in either responses to methacholine for *R*_RS_ or measurements of ED_200_ *R*_RS_ were observed between air‐exposed wild‐type and PAI‐1‐deficient mice (Fig. 6 and Table 1). Methacholine also significantly increased *R*_RS_ in O_3_‐exposed mice that were independent of genotype (Fig. 6). Dose–response curves to methacholine in O_3_‐exposed mice were shifted to the left when compared to genotype‐matched air‐exposed controls, a phenomenon demonstrated by a reduction in ED_200_ *R*_RS_ values for mice of both genotypes following O_3_ exposure (Table 1). However, a significant difference only existed between ED_200_ *R*_RS_ values for air‐ and O_3_‐exposed wild‐type mice. Nevertheless, responses to methacholine for *R*_RS_ were significantly greater in both O_3_‐exposed wild‐type mice and O_3_‐exposed PAI‐1‐deficient mice when compared to genotype‐matched air‐exposed controls. No genotype‐related differences in responses to methacholine for *R*_RS_ existed following O_3_ exposure.

## Discussion

To the best of our knowledge, we demonstrate for the first time that acute exposure to O_3_ increases total and active PAI‐1 in the epithelial lining fluid of the lungs (Fig. 1). In numerous animal models of lung injury, PAI‐1 is necessary for manifestation of many sequelae induced by acute exposure to O_3_, including migration of macrophages and neutrophils to air spaces, hyperpermeability of the alveolar‐capillary membrane, and AHR (Arndt et al. 2005, 2006; Kuramoto et al. 2009; Goolaerts et al. 2011; Wolthuis et al. 2011; Lee et al. 2012; Bhandary et al. 2015; Razvi et al. 2015; Tezuka et al. 2015; Liu et al. 2016). However, our results demonstrate that PAI‐1 does not contribute to the development of these aforementioned sequelae consequent to acute exposure to O_3_ (Figs. 3‐6).

Katre et al. (2011) and Kodavanti et al. (2011) reported that cyclic exposure to O_3_ increased PAI‐1 protein and mRNA expression, respectively, in rodent lung tissue. Consistent with these observations, we report that acute exposure to O_3_ increased BALF total and active PAI‐1 by five‐ and twelve‐fold, respectively (Fig. 1). Expression of PAI‐1 in the lungs is also increased *via* acid aspiration, antigen‐induced pulmonary injury, cigarette smoke, gram‐negative bacteria, hyperoxia, and mechanical ventilation with high tidal volumes (Barazzone et al. 1996; Oh et al. 2002; Kelly et al. 2005; Renckens et al. 2007; Allen et al. 2009; Kuramoto et al. 2009; Goolaerts et al. 2011; Wolthuis et al. 2011; Lee et al. 2012; Shetty et al. 2012; Tezuka et al. 2015). Therefore, increases in pulmonary expression of PAI‐1 subsequent to lung injury appear to be a nonspecific response, yet it is uncertain whether this phenomenon is beneficial or unfavorable. Indeed, PAI‐1 has been demonstrated to elicit both advantageous and adverse effects. For example, PAI‐1 facilitates the repair of lesions in airway and renal epithelial cells (Providence et al. 2000; Stevens et al. 2008). Alternatively, PAI‐1 can induce expression of pro‐inflammatory cytokines, migration of inflammatory leukocytes, and excessive deposition of collagen within the extracellular matrix (Eitzman et al. 1996; Bhandary et al. 2015; Tiwari et al. 2016). Thus, depending on the inciting stimulus, increases in PAI‐1 expression following lung injury may be beneficial, disadvantageous, or both.

In the absence of any inciting stimulus, expression of PAI‐1 mRNA within the lungs of C57BL/6 mice is localized to the airway and alveolar epithelium and pulmonary endothelial cells (Senoo et al. 2010; Wolthuis et al. 2011). Consistent with these observations, the apical cytoplasm of bronchiolar epithelial cells of air‐exposed wild‐type mice were strongly positive for PAI‐1 (Fig. 2A). Thus, airway epithelial cells are probably the most significant source of PAI‐1 that is found in the epithelial lining fluid of the lungs of air‐exposed wild‐type mice (Fig. 1). However, we cannot exclude the possibility that PAI‐1 detected in BALF of these animals is derived from other cell types within the lung that also express PAI‐1, including alveolar epithelial cells, fibroblasts, and pulmonary endothelial cells (Samad et al. 1994; Senoo et al. 2010; Wolthuis et al. 2011). It is also plausible that a fraction of PAI‐1 found in BALF is derived from the circulating blood.

Lung injury caused by LPS or mechanical ventilation with high tidal volumes leads to concomitant increases in BALF and lung tissue PAI‐1 (Arndt et al. 2005; Wolthuis et al. 2011). However, we observed opposing effects of O_3_ on BALF and lung tissue PAI‐1 in wild‐type mice. Despite the significant increase in BALF PAI‐1 induced by acute exposure to O_3_ (Fig. 1), PAI‐1 immunostaining was much weaker and more inconsistent within bronchiolar epithelial cells of O_3_‐ as compared to air‐exposed wild‐type mice (Fig. 2). These observations suggest that one or more phenomena may be occurring with regard to PAI‐1 expression in the airway epithelium following O_3_ exposure. First, O_3_ down‐regulates expression of PAI‐1 in airway epithelial cells. However, this scenario is questionable since previous reports demonstrate that O_3_ and other stimuli that cause lung injury, including antigen, cigarette smoke, gram‐negative bacteria, hyperoxia, and LPS, increase PAI‐1 expression in lung tissue (Barazzone et al. 1996; Oh et al. 2002; Arndt et al. 2005; Renckens et al. 2007; Katre et al. 2011; Kodavanti et al. 2011; Shetty et al. 2012). Second, O_3_ causes PAI‐1 release from airway epithelial cells at a rate greater than the airway epithelium can translate and/or transcribe PAI‐1 de novo following O_3_ exposure. This second scenario is more probable given the significant increase in BALF PAI‐1 caused by O_3_ exposure in wild‐type mice (Fig. 1). Nevertheless, the data that we currently present do not allow us to exclude the possibility that O_3_ actually suppresses PAI‐1 expression in the airway epithelium.

Plasminogen activator inhibitor‐1 has been previously shown to participate in inflammatory processes that arise as a result of lung injury by regulating expression of pro‐inflammatory cytokines and migration of inflammatory leukocytes. For example, Tiwari et al. (2016) report that PAI‐1 is necessary for maximal expression of KC and MIP‐2 following 20 weeks of exposure to cigarette smoke. In contrast, Wolthuis et al. (2011) demonstrate that PAI‐1 is necessary for the down‐regulation of IL‐6, KC, and MIP‐2 in response to ventilator‐induced lung injury. Similarly, Renckens et al. (Renckens et al. 2007) report that PAI‐1 suppresses pulmonary expression of IL‐6, KC, and MIP‐2 in mice intranasally inoculated with *Klebsiella pneumoniae*. Nevertheless, within this same report, Renckens et al. (2007) demonstrate that adenovirus‐mediated transfer of the PAI‐1 gene into the lungs of healthy mice increases expression of IL‐6, KC, and MIP‐2. Based on the ability of PAI‐1 to regulate expression of IL‐6, KC, and MIP‐2 in other animal models of lung disease, we hypothesized that PAI‐1 would regulate expression of these same cytokines following acute exposure to O_3_. Although O_3_ did increase BALF IL‐6, KC, and MIP‐2 in wild‐type and PAI‐1‐deficient mice (Fig. 3A–C), we did not observe, for the most part, any genotype‐related differences in any of these cytokines 4 or 24 h following cessation of exposure to O_3_. BALF MIP‐2, however, was significantly lower in PAI‐1‐deficient as compared to wild‐type mice 24 h following cessation of exposure to O_3_ (Fig. 3C). While statistically significant, we do not believe the decrease in BALF MIP‐2 observed in PAI‐1‐deficient mice is of any biological significance. We draw this conclusion based on our previously published data demonstrating the necessity of CXCR2, the receptor for KC and MIP‐2, for maximal neutrophil migration to the air spaces and airway responsiveness to methacholine following acute exposure to O_3_ (Johnston et al. 2005a; Konrad and Reutershan 2012). Thus, given that no genotype‐related differences in either the number of BALF neutrophils or airway responsiveness to methacholine were observed 24 h following cessation of O_3_ exposure (Figs. 3E and 6), we conclude that the statistically significant reduction in BALF MIP‐2 in PAI‐1‐deficient mice at this time interval is of no biological importance.

In addition to regulating expression of pro‐inflammatory cytokines, PAI‐1 regulates migration of inflammatory leukocytes to the lungs in response to lung injury. To that end, Bhandary et al. (2015) demonstrate that PAI‐1 is necessary for migration of macrophages to the air spaces following exposure to cigarette smoke. Furthermore, PAI‐1 is also required for the maximal migration of neutrophils to air spaces of mice exposed to cigarette smoke, gram‐negative bacteria, or LPS or mice mechanically ventilated with high tidal volumes (Arndt et al. 2005, 2006; Goolaerts et al. 2011; Wolthuis et al. 2011; Bhandary et al. 2015). Because PAI‐1 regulates migration of macrophages and neutrophils to air spaces following lung injury and because O_3_‐induced pulmonary inflammation is characterized, in part, by migration of macrophages and neutrophils to air spaces (Arndt et al. 2005, 2006; Johnston et al. 2005a; Wolthuis et al. 2011; Bhandary et al. 2015; Razvi et al. 2015), we hypothesized that macrophage and neutrophil migration to air spaces following exposure to O_3_ would be decreased in PAI‐1‐deficient mice. However, our data demonstrate that PAI‐1 deficiency had no effect on either the number of BALF macrophages and neutrophils (Fig. 3C and D) or perivascular inflammation in the lung (Fig. 4). Because BALF IL‐6, KC, and MIP‐2 were mostly unaffected by PAI‐1 deficiency following O_3_ exposure and because IL‐6, KC, and MIP‐2 are necessary for maximal neutrophil migration to air spaces following O_3_ exposure (Johnston et al. 2005a,b; Lang et al. 2008), we were not surprised that the number of BALF neutrophils were not different between wild‐type and PAI‐1‐deficient mice following O_3_ exposure (Fig. 3). Although our data with regard to the effect of PAI‐1 deficiency on macrophage and neutrophil recruitment to the lungs are inconsistent with the majority of studies examining the contribution of PAI‐1 to these phenomena within the lungs (Arndt et al. 2005, 2006; Goolaerts et al. 2011; Wolthuis et al. 2011; Bhandary et al. 2015), there are reports demonstrating that PAI‐1 has no effect on macrophage and neutrophil migration to the lungs following pulmonary injury (Rijneveld et al. 2003; Poggi et al. 2007; Allen et al. 2009). Thus, PAI‐1 is not always essential for inflammatory leukocyte recruitment to air spaces in response to lung injury.

Features of O_3_‐induced lung injury, including pulmonary vascular hyperpermeability that is characterized by an increase in BALF protein and airway epithelial cell desquamation, were not different between wild‐type and PAI‐1‐deficient mice, 4 and 24 h following acute exposure to O_3_ [Fig. 5 and (Scheel et al. 1959; Alpert et al. 1971; Bhalla et al. 1986)]. To the best of our knowledge, there have been only two studies examining the effect of PAI‐1 on the development of pulmonary vascular hyperpermeability (Barazzone et al. 1996; Goolaerts et al. 2011). In the study by Goolaerts et al. (2011), PAI‐1 facilitated the development of pulmonary vascular hyperpermeability in response to the intratracheal installation of *Pseudomonas aeruginosa* while Barazzone et al. (1996) reported that genetic deficiency of PAI‐1 failed to have an effect on pulmonary vascular permeability following 90 h of exposure to hyperoxia. PAI‐1 deficiency also failed to have any an effect on desquamation of airway epithelial cells 4 or 24 h following cessation of exposure to O_3_ (Fig. 5B). IL‐6 and CXCR2, the receptor for KC and MIP‐2, promote desquamation of airway epithelial cells following acute exposure to O_3_ (Johnston et al. 2005a; Lang et al. 2008; Konrad and Reutershan 2012). Because each of these cytokines were primarily unaffected in PAI‐1‐deficient mice following exposure to O_3_, it is not unexpected that PAI‐1 deficiency failed to have any impact on the desquamation of airway epithelial cells. Taken together, these results demonstrate that PAI‐1 has no effect on classic indices of lung injury induced by exposure to O_3_.

There was also no effect of PAI‐1 deficiency on AHR that developed 24 h following cessation of exposure to O_3_ (Fig. 6). In contrast, data from a number of investigators demonstrate that PAI‐1 is essential for the development of AHR following antigen sensitization and challenge (Kuramoto et al. 2009; Lee et al. 2012; Tezuka et al. 2015; Liu et al. 2016). Nevertheless, we were not surprised that there was no effect of PAI‐1 deficiency on the development of O_3_‐induced AHR. First, the development of O_3_‐induced AHR is dependent on CXCR2, the receptor for KC and MIP‐2 (Johnston et al. 2005a), and we observed, for the most part, no effect of PAI‐1 deficiency on BALF KC and MIP‐2 (Fig. 3B and C). Consequently, given the importance of KC and MIP‐2 to the development of AHR following acute exposure to O_3_ (Johnston et al. 2005a), we expected, in PAI‐1‐deficient mice, any O_3_‐induced changes in airway responsiveness to methacholine to correlate with any O_3_‐induced changes in BALF KC and MIP‐2. Second, features of airway remodeling are nonexistent in lungs of wild‐type mice 24 h following cessation of acute exposure to O_3_ (2.5 ppm for 3 h) (Triantaphyllopoulos et al. 2011). Airway remodeling, which is typified, in part, by subepithelial fibrosis, goblet cell hyperplasia, and hypertrophy of airway smooth muscle cells, leads to AHR (Homer and Elias 2005). Chronic antigen challenge causes airway remodeling and AHR in wild‐type mice, and PAI‐1 deficiency reduces the severity of these sequelae (Kuramoto et al. 2009; Lee et al. 2012; Tezuka et al. 2015; Liu et al. 2016). Thus, to date, the ability of PAI‐1 to contribute to the development of AHR appears to be mechanistically coupled to the ability of PAI‐1 to promote features of airway remodeling. Because acute exposure to O_3_ fails to elicit airway remodeling (Triantaphyllopoulos et al. 2011), this may explain why we failed to observe an effect of PAI‐1 deficiency on the development of O_3_‐induced AHR.

In a majority of the studies examining the contribution of PAI‐1 to the regulation of pro‐inflammatory cytokine expression, migration of inflammatory leukocytes, and AHR following exposure to diverse injurious stimuli, PAI‐1 was demonstrated to contribute to these aforementioned sequelae. However, PAI‐1 did not make a significant contribution to these same sequelae following acute exposure to O_3_ (Figs. 3, 4, 5, 6). There are a number of plausible reasons that may explain why we failed to observe an effect of PAI‐1 deficiency on O_3_‐induced pulmonary pathology. First, the ability of PAI‐1 to induce these sequelae may be stimulus specific, which is an observation that is consistently observed for IL‐6 (Cenci et al. 2001; Johnston et al. 2005b). Second, the time intervals at which we measured our outcome indicators may not be capturing an effect of PAI‐1 deficiency. For example, interruption of IL‐6 signaling has an effect on desquamation of airway epithelial cells at 24 h but not 4 h following cessation of exposure to O_3_ (Johnston et al. 2005b; Lang et al. 2008). Thus, if we examine these same indices at other time intervals (e.g., 2, 8, or 36 h) following cessation of exposure, we may observe an effect of PAI‐1‐deficiency. Third, duration of exposure to the inciting stimulus may be important to observe an effect of PAI‐1 deficiency on our outcome indicators. In many of the aforementioned studies where PAI‐1 was demonstrated to have an effect, exposure to the inciting stimulus was for at least 5 h or more, and in some instances, lasted for several days to weeks. Pulmonary responses induced by exposure to O_3_ acutely (2 ppm for 3 h) as compared to subacutely (0.3 ppm for 72 h) are controlled by different genetic loci (Kleeberger et al. 1993). Therefore, it is entirely plausible that PAI‐1 regulates responses to subacute as compared to acute O_3_ exposure.

In conclusion, we demonstrate that there are robust increases in PAI‐1 in the epithelial lining fluid of the lungs following acute exposure to O_3_. Despite these increases in PAI‐1 within the lungs, PAI‐1 did not functionally contribute to any aspect of the pulmonary pathology induced by acute exposure to O_3_, including migration of inflammatory leukocytes to air spaces, pulmonary vascular hyperpermeability, and AHR. Nevertheless, these results do not exclude the possibility that PAI‐1 may contribute to the pulmonary pathology induced by subacute or chronic exposure to O_3_.

## Conflict of Interest

None declared.
